# The impact of pre and perinatal lifestyle factors on child long term health and social outcomes: a systematic review

**DOI:** 10.1186/s13561-018-0186-6

**Published:** 2018-01-24

**Authors:** Kerry Bell, Belen Corbacho, Sarah Ronaldson, Gerry Richardson, David Torgerson, Michael Robling, on behalf of the Building Blocks trial group

**Affiliations:** 1 York Trials Unit, Department of Health Sciences, University of York, ARRC Building, York, YO10 5DD UK; 2 Centre for Health Economics, University of York, York, YO10 5DD UK; 3 Centre for Trials Research, Cardiff University, Cardiff, CF14 4YS Wales

**Keywords:** Home visitation, Birth weight, Smoking, Pregnancy, Child outcomes, Education, Health

## Abstract

To understand the full extent of the impact of a trial, it is important to consider the long-term consequences of outcomes beyond the trial follow-up period, especially for early year’s interventions. A systematic review of the literature associated with the long-term consequences of four key outcomes from the Building Blocks trial, specifically, low birth weight, smoking during pregnancy, interval to subsequent pregnancy and A&E attendance or inpatient admission was conducted. These factors were guided by the funders, the Department of Health, as being of particular interest in the UK context. Relevant studies were identified from a number of sources including large databases, reference checking and citation searching. The search yielded 3665 papers, 43 of which were considered appropriate for inclusion. Of these, 29 were relating to smoking during pregnancy, 13 to low birth weight, 0 to A&E attendances during early childhood and 1 to short (< 2 years) interval to subsequent pregnancy. Consistent associations were found between maternal smoking during pregnancy and the effects this has on children’s health, educational attainment and likelihood of engaging in problem behaviour and criminal activity in later life. Low birth weight was also found to impact on children’s long-term health and cognitive development. Subsequent pregnancies within two years of the previous birth were linked with increased likelihood of pre-term birth and neonatal death. Only minimal evidence was identified regarding the consequences of a short interval to second pregnancy and of child A&E and outpatient attendances. Given that these outcomes have been identified by the UK Department of Health as of particular interest for UK benefit, investment of research in these areas is recommended to establish a clearer picture of both short and long-term consequences.

## Review

A key short coming within many randomised controlled trials (RCTs) is their relatively short term follow-up. This is a particular problem for early interventions aimed at pregnant women or young children where we might expect some major impacts on children’s health and welfare to only become evident some years after an intervention. Consequently, many economic evaluations based on RCT data undertake modelling exercises to assess the cost effectiveness of an intervention making assumptions about long term treatment effects. A key issue underpinning such modelling is whether or not proximal outcomes observed in a RCT, and which are often proxy measures, are causally linked to more distal outcomes among children at a later age. For instance, we can be pretty confident that cessation of smoking among pregnant women (a proximal and proxy outcome) will be linked to a future reduction in cancer among the mothers and increases in birthweight among babies. However, there is a greater uncertainty as to whether close mother child bonding will lead to better outcomes in terms of educational achievement lower rates of delinquency and overall increases in future wellbeing.

Although longitudinal outcomes can carry substantial weight in decisions of cost-effectiveness, randomised controlled trials are often limited in their ability to measure outcomes over a longer time frame due to feasibility and funding restraints [1]. In these situations, extrapolation modelling can provide decision makers with estimates of the potential long-term consequences associated with the outcomes of a trial by linking information from the wider literature to trial outcome data. The purpose of this being to provide decision makers with a more complete picture of the costs and outcomes of the intervention than the trial alone would do. This type of evidence synthesis and decision modelling are a central process of Health Technology Assessment (HTA), and represent a crucial role in the NICE appraisal process [2].

Due to the potential influence on long-term development, interventions in childhood are particularly linked to long-term consequences both in terms of future benefits and future cost-savings [3–5]. Recognising the importance of linking the outcomes of childhood interventions to long-term developmental trajectories, large scale extrapolation models have been developed both in the United States (US) by the Washington State Institute for Public Policy (WSIPP) [6] and in the United Kingdom (UK) by the Social Research Unit (SRU) [7]. The present work formed part of a comprehensive economic evaluation of Building Blocks, a randomised controlled trial investigating the effectiveness and cost-effectiveness of the provision to first-time teenage mothers of the Family Nurse Partnership (FNP) programme on improving specified infant and maternal outcomes [8]. The trial provided comprehensive data for up to 24 months after birth in terms of the effect on costs and outcomes. However, given that childhood interventions often deliver effects beyond the exploratory period, in this case approximately 30 months, an extrapolation exercise was planned to identify the potential wider and longer-term consequences associated with modifying the primary outcomes of the trial. Existing models such as those constructed by WSIPP and SRU which were designed to predict the potential long-term impact of competing investment options for child wellbeing, as well as the costs and economic returns of interventions [6], use educational attainment and problem behaviour and criminal activity as a basis of their models hence these, as well as health, were considered as the most appropriate outcomes on which to base the Building Blocks extrapolation exercise.

The present review examines the literature associated with the long-term consequences of four key outcome measures from the Building Blocks trial. In commissioning the trial, the Department of Health Policy Research Programme that funded the trial specified inclusion of prenatal tobacco use, childhood injuries requiring emergency department attendance or admission, and inter-birth interval as primary outcome measures that were considered on the basis of previous trials to be modifiable by FNP. Having being recommended to the funders by an advisory committee including representatives of the programme, birthweight was also specified as a primary outcome as a policy-relevant and readily measurable outcome that was applicable to all trial participants.

Specifically, the review aimed to identify relevant longitudinal studies relating to the longer term effects of:

- Maternal smoking during pregnancy on the child.
- Infant low birth weight.
- Short-interval to subsequent pregnancy (defined as less than two years).
- Childhood A&E attendances and inpatient admissions.

## Methods

### Search strategy

The databases MEDLINE and MEDLINE In Process & Other Non-Indexed Citations (Ovid), British Education Index (ProQuest), Criminal Justice Abstracts (EBSCO), ERIC (ProQuest), PsycINFO (Ovid), Social Policy and Practice (Ovid) and Social Science Citation Index (Web of Knowledge), covering literature published to 2012 (A&E attendances to 2013) were searched using a pre-specified search strategy. The observational study design filter created by SIGN was used and adapted for these searches [9]. In addition to searching these databases, reference checking and citation searching from identified papers was also carried out.

Potentially relevant articles were retrieved and saved in an EndNote library. The search strategy was restricted to include only studies conducted in the four UK constituent countries (England, Scotland, Wales and Northern Ireland). These studies were then assessed for potential inclusion in the review based on a strict set of inclusion and exclusion criteria.

### Inclusion and exclusion criteria

All longitudinal prospective or retrospective design, relating to the primary trial outcomes (birth weight, prenatal tobacco use, emergency attendances and inpatient admissions, and second pregnancy within two years of first birth), measuring child health/well-being/criminality/education were considered. Due to time restraints for the delivery of the review within the context of the wider Building Blocks trial, and to ensure generalisability to a UK health context, only studies set within the UK were included.

Studies were excluded if the outcome measure of interest was not being investigated as the exposure variable. Study outcomes were not limited by time, i.e. studies could measure outcomes at later points in childhood, adolescence and adulthood.

### Assessment of studies

**Fig. 1:**
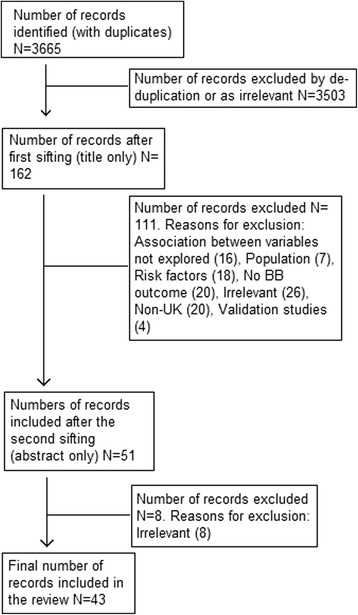
Flow chart showing the selection process for the systematic review

Study selection took a step-wise approach. Article titles and, where available, abstracts were screened to determine whether they fulfilled eligibility criteria. Articles not immediately meeting the inclusion criteria were rejected and the reasons for exclusion recorded. Where abstracts were unavailable, full papers were retrieved for consideration. Papers meeting the full inclusion criteria were retrieved for detailed assessment. The first 10% of citations were screened independently by both the primary reviewer and a secondary reviewer to minimise the risk of bias or errors. Any disagreements were resolved via discussion between the two reviewers. The inclusion strategy was then definitively established and the primary reviewer completed the remaining 90% of the papers. A flow chart showing the number of studies remaining at each stage was used to document this selection process (Fig. 1).

Information on study design, participant characteristics, outcomes, length of follow-up, method of analysis and main findings were extracted.

### Quality assessment

Critical appraisal of identified studies was undertaken with the aid of a known checklist. Although several tools exist, no single tool has been adopted universally to assess quality in non-randomised studies [10]. The existing tools have been systematically reviewed [11], resulting in six tools being identified as useful for quality assessment, though all of which requiring a level of adjustment depending on the research questions. For the present review, relevant articles were evaluated using an adapted version of the Effective Public Health Practice Project (EPHPP) ‘Quality Assessment Tool for Quantitative Studies’ [12], as identified as a useful tool in the systematic review. This tool considers the appropriateness of the study design to the research question, risk of bias, choice of outcome measure, analytical methods, quality of reporting, quality of the intervention and generalisability.

### Data analysis

A high degree of heterogeneity was identified between the studies meeting the inclusion criteria for the review in terms of study type, sampling and methods used. The results are discussed through means of a narrative synthesis, which highlights the potential long-term benefits that may arise from improvements in these early outcomes. The results are summarised according to the outcome measure assessed.

## Results

The search identified a total of 3665 records of which 3503 were excluded on the basis of title and abstract, leaving a total of 162 for retrieval and full study assessment. Of these, 43 records were deemed suitable, fulfilling the inclusion criteria for the review.

Of the 43 studies retrieved 29 concerned smoking related outcomes, 13 concerned outcomes associated with low birth weight and 1 discussed the outcomes associated with short inter-pregnancy interval.

As per the inclusion criteria, all studies were conducted within a UK context, though one study included comparative data from the Czech Republic [13]. Studies comprised varying sample sizes and examined a vast range of outcomes. The majority of the studies were prospective longitudinal cohort studies (*n* = 33), though 10 retrospective/case-control studies also met the inclusion criteria.

The included studies were largely of moderate or high quality, though two studies did not meet the minimum criteria outlined in the quality checklist and thus were deemed low quality.

### Maternal smoking and child outcomes

#### Health

Maternal smoking during pregnancy was linked to a wide range of both childhood and adulthood health outcomes. Table 1 summarises the findings.

**Table 1:** Maternal smoking during pregnancy and child health outcomes

Study	Aim	Methods	Outcome measure	Findings	Notes	Quality assessment
Blair et al., 1996 [26]	To investigate the effect of exposure to tobacco smoke on the sudden infant death syndrome	Two year population based case-control study Participants: 195 babies who died and 780 matched controls	SIDS	A dose response was associated with exposure to tobacco smoke. Maternal smoking during pregnancy was significantly related to SIDS (OR = 2.10 [95% CI:1.24-3.54]) in the multivariate analysis. After adjustment, paternal smoking had an additional independent effect (OR = 2.50 [95% CI: 1.48- 4.22])	Adjusted for a good amount of covariates.	Moderate
Fertig, 2010 [52]	To examine the importance of selection on the effect of prenatal smoking by using three British cohorts	Data from 3 UK birth cohort studies used providing a large data set of 45,400 participants.	Birth weight	The effect of prenatal smoking in 2000 on low birth weight is over 50% greater than in 1958 and is approximately double with respect to the probability of a low birth weight birth conditional on gestation. Selection could explain as much as 50% of the current association between prenatal smoking and the probability of low birth weight birth.	Adjusted for a good amount of covariates.	Strong
Golding et al., 1990 [14]	Association between child cancer and factors during pregnancy, labour and delivery and other maternal aspects.	Case-control study of 132 children. Development of childhood cancer was recorded.	Child cancer	Childhood cancer was associated with antenatal smoking (OR = 2.69 [95%CI:1.05-6.89]). Logistic regression showed independent relationship between childhood cancer and maternal smoking (OR = 2.5 [95% CI:1.20-5.08])	Some covariates accounted for.	Moderate
Hawkins et al., 2009 [18]	Association between risk factors (including birth weight and smoking during pregnancy) obesity.	Prospective cohort study using data from the Millennium Cohort Study (*n* = 13,188)	Childhood obesity	Early childhood obesity was associated with maternal smoking during pregnancy (1-9 cigarettes daily: OR = 1.34 [1.17-1.54] fully adjusted; 10-19 cigarettes: OR = 1.49 [1.26-1.75] fully adjusted).	Adjusted for a good amount of covariates.	Strong
Henderson et al., 2001 [13]	Association between smoking during pregnancy, environmental tobacco smoke (ETS) exposure and wheezing illness of infants of 6 months old	Longitudinal cohort studies from the UK and Czech Republic (*n* = 14,269).	Wheeze	In the UK, infant wheeze was significantly associated with maternal smoking during pregnancy OR = 1.30 [95% CI: 1.09-1.56] adjusted).	Adjusted for a good amount of covariates.	Moderate
Koshy et al., 2011 [19]	Association between children’s weight and height and cigarette smoke exposure during mothers’ pregnancy	Use 2 UK cross-sectional surveys from 1998 and 2006 (*n* = 3038)	Childhood obesity	Smoking during pregnancy was associated with an increase in the likelihood of obesity in children (OR = 1.61[95% CI: 1.19–2.18])	Some covariates included.	Moderate
Larsson & Montgomery (2011) [28]	To assess the association between smoking during pregnancy and poorer motor competence among offspring	Longitudinal study of 13,207 families in GB followed up to age 11.	Hand control and coordination assessed using known measures.	After adjustment, heavy smoking during pregnancy was significantly associated with poorer performance in PUM (picking up matches) task for the non-dominant hand in both boys (Coeff = 1.474 [95% CI: 0.47-2.48 *p* = .004] and girls (Coeff = 1.203 [95% CI: 0.15-2.26 *p* = .026]). It also negatively affected boys’ performance in CD (copying design test score) (Coeff = −0.185 [95% CI:-0.32 - -0.05 *p* = .006]).	Good covariates	Moderate
Little et al. 2004 [27]	Association between smoking and orofacial clefts	Case-control study of 438 children from England and Scotland.	Orofacial clefts	Maternal smoking during pregnancy had a positive association with cleft lip with or without cleft palate (CL+/-P)(OR = 1.9 [95% CI:1.1-3.1] adjusted), and cleft palate (CP) (OR = 2.3 [95% CI:1.3-4.1] adjusted). A dose-response was observed for both CL+/-P (*p* value = 0.012) and CP (*p* value = 0.004). Passive smoking of mothers also had weak effect.	Adjusted for a good amount of covariates.	Moderate
Pang et al. 2003 [53]	Association between parental preconceptional smoking and maternal smoking in pregnancy, and risk of developing cancer in childhood	UK based case control study using children diagnosed with malignancy or CNS tumour under 15 years (*n* = 3838) and matched controls (*n* = 7629).	Childhood cancer	Significant monotonic decreasing trends in risk were found in relation to the amount of cigarettes smoked by the mother during pregnancy for all child cancers, leukaemia, lymphoma, CNS tumours and other solid tumours (*p* < 0.001, *p* = 0.03, *p* = 0.01 and *P* = 0.03 respectively), with ORs statistically significantly below 1 among heavy smokers. For primitive neuroectodermal tumours the OR was 0.55 (*P* = 0.01).	Some covariates included.	Strong
Power et al. 2010 [20]	Association between maternal smoking during pregnancy and risk factors for CVD	Prospective UK cohort study following members up to age 45 years (*n* = 8815).	Risk factors of CVD in adulthood	Maternal smoking during pregnancy was associated with an increased likelihood of obesity in adult offspring classified by BMI (OR = 1.40 [95% CI: 1.25–1.56]) and high waist circumference (OR = 1.32 [95% CI: 1.19–1.47])	Adjusted for a good amount of covariates.	Strong
Power et al. 2003 [22]	To investigate growth trajectories and predictive factors for those with low birth weight and high adult BMI	Birth cohort study followed up to age 33. Full data available for 7017 participants.	Adulthood obesity	Maternal smoking during pregnancy was associated with an increased likelihood of obesity in adult offspring classified by BMI for both males (OR = 1.79 [95% CI: 1.37-2.29] and females (OR = 2.27 [95% CI: 1.79-2.86]).	Some covariates adjusted for.	Strong
Power and Jefferies 2002 [21]	Association between maternal smoking during pregnancy and obesity risk through childhood to age 33	Prospective GB cohort study of 5839 born in 1958. Assessed obesity status (BMI) at age 33.	Adulthood obesity	Maternal smoking during pregnancy was associated with an increased likelihood of obesity in adult offspring classified by BMI for both males (OR = 1.56 [95% CI: 1.22-2.00] and females (OR = 1.41 [95% CI: 1.12-1.79].	Good range of covariates.	Strong
Ramadas et al. 2007 [15]	1. Association between the IL1RN gene polymorphisms with asthma; and 2. association between the gene (IL1RN)-environment (smoke exposure) interactions and asthma	UK based prospective cohort study. Outcome measure: Asthma, airway obstruction and BHR	Asthma	The rs2234678 genotype GG was significantly associated with repeated measurements of asthma in children of mothers who smoked during pregnancy (ETS-2 group: OR 4.43, CI 1.62–12.1, *p* = 0.0037) but not in children without maternal smoking exposure during pregnancy (ETS-0 or ETS-1). This suggests that exposure to maternal smoking may be more detrimental to some children than others.	Some covariates adjusted for.	Moderate
Sadeghnejad et al. 2008 [16]	To investigate whether there is a combined effect of interleukin-I3 gene polymorphisms and tobacco smoke on persistent childhood wheezing and asthma	UK based cohort study followed up to age 10 (*n* = 791) Outcomes were wheezing and persistent childhood asthma	Wheezing and asthma	Maternal smoking during pregnancy was associated with early onset persistent wheeze (OR = 2.93, *p* < 0.0001). However, the effect of maternal smoking during pregnancy was stronger in children with certain genetic features (OR = 5.58 and OR =1.29, respectively; p for interaction = 0.014). When analyzing asthma instead of wheezing, the interaction was statistically significant (*p* = 0.03) for persistent asthma. Children with a CCG/CCG haplotype pair had an OR of 5.57 (95% CI 2.13 to 14.63, *p* = 0.0005) for ETS-2 on persistent asthma. For subjects with haplotype pairs other than CCG/CCG, the OR was 1.32 (95% CI 0.57 to 3.04, *p* = 0.587).	Some covariates adjusted for.	Moderate
Severson et al. 1993 [54]	Association between parental smoking and alcohol consumption and childhood AML.	Case control study in the US and Canada.	Childhood cancer (Acute Myeloid Leukemia (AML))	No statistically significant associations were found for maternal cigarette smoking when exposures were restricted to the month immediately preceding pregnancy; the first, second, or third trimester of pregnancy; or during the time the mother was nursing the index child.	Some covariates adjusted for.	Moderate
Sorahan et al. 1995 [55]	Association between any childhood cancer and consumption of alcohol and tobacco.	Case-control study in England and Wales.	Childhood cancer	There was no association between maternal smoking and childhood cancer (*P* = 0.602).	Only class, maternal and paternal age controlled for.	Moderate
Sorahan and Lancashire 2004 [56]	Relation between parental cigarette smoking and hepatoblastoma	Case-control study in UK. 43 cases of hepatoblastoma and 5777 controls	Childhood cancer	Positive associations were found between hepatoblastoma risks and both maternal and paternal smoking. The largest relative risk is shown in the fuller model for both parents being smokers (RR = 2.69, *P* < 0.05, 95% CI 1.18–6.13).	Good range of covariates.	Moderate
Strachan et al. 1996 [17]	The relationship between incidence of wheezing illness from birth to age 33 and perinatal, medical, social, environmental, and lifestyle factors	Prospective longitudinal study across the UK. 18,559 participants followed to age 33	Asthma and wheeze	Maternal smoking during pregnancy was associated with increased incidence of childhood wheezing (OR = 1.72 [95% CI: 1.11-2.67]), when compared to cohort members whose mother never smoked. Birth order, birth weight and birth weight for gestation were not significant independent risk factors.	Good range of covariates.	Strong
Thomas et al. 2007 [23]	To explore how prenatal exposures known to be associated with low birth weight effect glucose metabolism in midlife.	UK cohort study of 7518 cohort members born in 1958.	Adult metabolism (diabetes)	No association was found between maternal smoking and blood glucose levels in offspring after accounting for birth weight for gestational age (BGA) and adult adiposity.	Some covariates adjusted for.	Strong
Toschke et al. 2007 [24]	To look at the association between maternal smoking and type 2 diabetes.	GB prospective cohort study. 5214 cohort members from NCDS and 6069 from BCS70.	Diabetes	No association between diabetes and postnatal maternal smoking was observed.	Good range of covariates	Moderate
Toschke et al. 2003 [25]	Association between smoking in pregnancy and appetite control in offspring	GB prospective cohort study 10,557 cohort members born 1958	Appetite control	An association was found between maternal smoking during pregnancy and offspring appetite control in adulthood (OR = 1.22 [95% CI: 1.01-1.48]).	Some covariates adjusted for.	Moderate

Of five studies exploring the association between prenatal exposure to maternal smoking and incidence of childhood cancer, only one reported significantly elevated odds of development [14]. This study was unique in the sense that it analysed the impact based on all types of childhood cancer, in contrast to the other four which focused on only specific types of cancer. Whilst this aspect of the design may be beneficial to assessing the relationship between smoking exposure and the broad spectrum of cancer diseases, the study was largely hindered by a very small case-control based sample size (*n* = 132), matched on only a narrow range of potentially confounding variables. Given the limitations of this design and the high correlation between the other four studies identified, there appears to be little evidence in a UK setting supporting a link between maternal smoking and subsequent incidence of childhood cancers.

Wheeze and asthma related health problems were consistently associated with smoking during pregnancy [13, 15–17], with all four studies investigating this outcome reporting a significant association (OR 1.3-4.43). All four studies were deemed of moderate to high quality, employing large, generalizable samples and accounting for a good range of covariates in statistical models.

The most prominent health concern associated with maternal prenatal smoking was weight related problems, which were noted not only in childhood but also throughout adulthood. Eight studies engaged this as an outcome measure, two of which looked at child outcomes [18, 19] with the remaining six focusing on adult outcomes [20–25]. Children of prenatal smokers were between 1.23 and 1.49 times more likely than children of non-smokers to develop childhood obesity, according to the number of cigarettes mothers smoked per day [18, 19]. The probability of developing obesity in adulthood was slightly more pronounced with similar increases in the likelihood being found across the 3 studies using this outcome (OR 1.4-2.27) [20–22]. Additionally children of prenatal smokers were also more likely to experience poor appetite control in adulthood (likely to facilitate obesity) [25], and may be more likely to develop diabetes [23], though this specific association is questionable and inconsistent across studies [24]. Given the vast health related costs associated with weight related problems, this finding presents a clear example of how a reduction in maternal prenatal smoking could be vastly beneficial and cost saving over time.

Links were also identified with Sudden Infant Death Syndrome (SIDS) [26], orofacial abnormalities such as cleft palate [27], and neurological functioning assessed through measures of motor control [28], emphasising the breadth of potential health benefits that could be achieved by reducing maternal smoking during pregnancy.

#### Cognitive development and educational attainment

Table 2 presents the findings relating to maternal smoking and child cognitive and educational outcomes. No association was identified between maternal prenatal smoking and children’s academic attainment measured through test-scores [29]. However, two studies did report a significant association with problematic behaviour in school aged children [30, 31], which may affect learning. Increases in the likelihood of children engaging in problematic behaviour ranged from 1.17 times more likely to 1.80 times more likely depending on the type of behaviour measured and the quantity of cigarettes the mother had smoked during pregnancy. However, both of these studies lacked a rigorous selection of potential confounders in the analysis. Additionally, the final study employing this outcome reported no significant association after covariates had been adjusted for [32].

**Table 2:** Maternal smoking during pregnancy and child cognitive/educational outcomes

Study	Aim	Methods	Outcome measure	Findings	Notes	Quality assessment
Brion et al., (2010) [30]	To assess the association between maternal prenatal smoking and child psychological problems.	Prospective cohort study in 3 health districts in England and Brazil. *N* = 6735 in England, 509 children in Brazil	Behavioural outcomes measured by Strengths and Difficulties Questionnaire (England) or Child Behaviour Checklist (Brazil) around age 4	In the UK cohort maternal smoking was significantly associated with hyperactivity/attention problems (OR = 1.17 [95% CI 1.04-1.31]), and peer social problems (OR = 1.24 [95% CI 1.1-1.4]). Smoking was also associated with conduct/externalizing problems (OR = 1.24 [95% CI:1.07-1.46])	Adjusted for good range of covariates included paternal smoking	Strong
Collins et al., (2007) [29]	To assess the association between prenatal tobacco exposure and child academic achievement.	Longitudinal analysis of 6390 mother-child pairs across the UK.	Adolescent offspring academic achievement measured through pass/fail on O-level (GCSE equivalent) and A-level at ages 16 and 18 respectively.	Prenatal exposure had no significant effects on test failure in adolescence.	Some covariates accounted for	Moderate
Hutchinson et al., (2010) [31]	Associations between maternal smoking in pregnancy and child behaviour.	Prospective cohort study of 13,778 families across the UK (MCS) followed from birth	Children’s conduct and hyperactivity/inattention problems measured by the SDQ at age 3 years.	After adjustment, for boys, mothers’ persistent smoking in pregnancy was significantly associated with conduct problems (OR = 1.44 [95% CI: 1.01-2.06] for light smoker; OR = 1.80 [95% CI: 1.28-2.54] for heavy smoker) and hyperactivity-inattention problem (OR = 1.56 [95% CI: 1.12-2.15] for light smoker; OR = 1.62 [95% CI: 1.13-2.33] for heavy smoker).	Good range of covariates	Strong
Maughan et al., (2004) [32]	To explore the association between prenatal smoking and early childhood behaviour	Longitudinal study of 1116 families in England and Wales.	Children’s conduct problems at age 5 and 7 years were assessed using the CBCL measures.	No significant association between maternal smoking and child behaviour.	Some covariates	Moderate

#### Problem behaviour and criminal activity

As shown in Table 3, three studies reporting on problem behaviour and criminal activity or anti-social behaviour measures during late childhood through to adulthood met the criteria for inclusion. Although no significant association was found between maternal smoking and child smoking and alcohol use at age 10 (Macleod et al., 2008) and child antisocial behaviour at age 16 [33], children of prenatal smokers were found to be more likely to report convictions for criminal activity in adulthood (OR 1.4-1.8), with female children being more affected than male children [34]. Although these findings are drawn from only 1 study, this was deemed high quality research boasting a vast sample size (*n* = 16,401), where a diverse range of covariates were included in statistical analyses, including maternal depression, a known risk factor for problematic behaviour and criminal activity in children [35–37].

**Table 3:** Maternal smoking during pregnancy and child criminal/anti-social activity in adolescence and adulthood

Study	Aim	Methods	Outcome measure	Findings	Notes	Quality assessment
Hay et al., (2010) [33]	To examine the links between exposure to maternal depression in pregnancy and antisocial outcomes in children.	Longitudinal study of 120 families in Britain. Families were followed until the child was around 16 years.	Incidence of arrests and DSM diagnoses.	Antenatal exposure to cigarette smoking did not predict antisocial outcomes for children.	Adjusted for a wide range of covariates	Moderate
Macleod et al., (2008) [57]	To estimate the prevalence of alcohol and tobacco use among children	Birth cohort study in England (*n* = 6895, 3410 male)	Measured self-reported use of tobacco and alcohol at age 10.	After adjusting for potential covariates no significant association was found between maternal smoking and child tobacco and alcohol use.	Good range of covariates. 10 years old likely too young for outcome measure	Moderate
Murray et al., (2010) [34]	To identify early predictors of conduct problems and crime	Large UK cohort study (*n* = 16,401) followed up to age 34 years	Child conduct problems at age 10 measured using parent-rated Rutter A2 scale. Convictions were self-reported at ages 30 and 34 years.	In fully adjusted models, maternal smoking during pregnancy was significantly associated with conduct problems at age 10 (partial OR = 1.8 [95% CI: 1.3-2.5] for girls; Partial OR = 1.7 [95% CI: 1.4-2.2] for boys) and convictions in adulthood (partial OR = 1.8 [95% CI: 1.2-2.7] for girls; partial OR = 1.4 [95% CI: 1.1-1.7] for boys).	Adjusted for a wide range of covariates	Strong

### Low infant birth weight and child outcomes

#### Health

Table 4 presents the findings relating to low birth weight and health. Data were extracted from 9 studies exploring the effects of low birth weight on future health outcomes. Three of these were prospective cohort studies representative of the entire British population [38–40], four longitudinal [41–44] and two cross- sectional [45, 46] studies.

**Table 4:** Low birth weight and childhood health outcomes

Study	Aim	Methods	Outcome measure	Results	Notes	Quality assessment
Annesi-Maesano et al., 2001 [38]	To explore how in utero and perinatal factors and health outcomes affect the development and severity of asthma in childhood	Prospective cohort study. 4065 children and 2583 mothers. Mean age among cohort mothers was 31.0 ± 3.3 years.	Childhood asthma	Low birth weight (< 2.5 kg) was associated with child asthma (OR = 1.57 [95% CI: 1.10- 2.25])	Good range of covariates	Strong
Davies et al. 2004 [45]	Association between birth weight and adult total cholesterol concentration (TC)	Cross-sectional 1994-1996 18,286 men and 7557 women. British Telecom employees The mean age is 38.9 for men and 36 for women	Adult cholesterol	After adjustment, in men a − 0.09 mmol/L reduction in TC was observed per 1 kg increase in birth weight (95% CI, − 0.11 to − 0.06 mmol/L; *P* < 0.001); in women, a − 0.006 mmol/L reduction in TC was observed per 1 kg increase in birth weight (95% CI, − 0.04 to 0.03; *P* = 0.8).	Birth weight and TC association may be dependent on sex. Adjusted for a good amount of covariates	Moderate
Gale and Martyn 2004 [39]	Association between birth weight and risk of psychological distress and depression	Prospective cohort study 5187 participants included for the 16-year follow-up; 8292 for the 26-year follow-up	Adult depression	Low birth weight < =2.5 kg was associated with depression in women at age 26 (OR = 1.3 [95% CI:0.9-1.8]) and men at age 26 years (OR = 1.6 [95% CI:1.1-2.3]).	Adjusted for a good amount of covariates	Strong
Law et al. 1993 [40]	Association between low birth weight and high blood pressure	Longitudinal study 1895 children (0-10 years) and 1231 men and women aged 59-71 years.	Childhood and adult blood pressure	Every kg of birth weight increase was associated with 2.8 mmHg (95% CI: 1.4-4.1) decrease in blood pressure at the age of 4, 4.0 mmHg (95% CI: 1.5-6.5) decrease at the age of 64, and 5.2 mmHg (95% CI: 1.8-8.6) decrease for the age of 64 to 71	Blood pressure as a proxy of hypertension	Strong
Moore 2005 [46]	To identify the incidence and characteristics of preventable childhood deaths.	Retrospective survey 34 childhood preventable deaths. City of Wolverhampton, UK Mother’s age < 20 years (33%)	Childhood death	Preventable deaths were associated with low (2933 g) birth weight (*p* < 0.001).		Weak
Orfei et al. 2008 [58]	Association between adult lung function and birth weight, postnatal growth and early air-pollution exposure	Data drawn from 2 UK cohort studies (*n* = 3262 and 9377). Lung function (forced expiratory volume in 1 s (FEV1), and forced vital capacity (FVC)). The 1946 cohort was assessed for lung function at age 43 years; the 1958 cohort was assessed at age 44-45 years.	Lung function	When the two cohorts were pooled and mutually adjusted, 1 SD increase in birth weight was associated with 30.4 ml increase in FEV1 (95% CI: 16.1-44.8) and 26.9 ml increase in FVC (95% CI: 8.0-46.0).	Adjusted for a good amount of covariates.	Strong
Pearce et al. 2012 [41]	Direct and indirect associations between foetal, infancy and adult risk factors and fibrinogen levels	Prospective study 394 singleton study members Newcastle upon Tyne, UK	Adult fibrinogen level (a risk factor for CVD)	No significant association was found between standardised birth weight and adult plasma fibrinogen levels (Beta-coefficient = − 0.03, 95% CI: − 0.01-0.001, *p* = 0.34, unadjusted)		Moderate
Riordan et al. 2006 [42]	The association between perinatal circumstances and subsequent young adult suicide	Birth cohort study 1,061,830 participants birth between 1 Jan 1969 - 31 Dec 1986	Adult suicide	Individuals of low birth weight (< 2500 g), when compared with the reference group (3250–3749 g) were at higher risk of suicide (HR = 1.35, 95% CI 1.05–1.72) and at higher risk of death from other causes (HR = 1.41, 95% CI 1.24–1.61).		Strong
Robertson and Harrild 2010 [43]	Association between maternal and neonatal risk factors and type 1 diabetes in children under 15 years old	361 case children and 1083 controls 1972-2005 Scottish Study	Childhood diabetes	The risk of development childhood type 1 diabetes was not associated with birth weight (OR 0.66 CI95% 0.34 to 1.28; *p* = 0.22 adjusted).		Moderate
Smith et al. 2007 [44]	The relation between complications in a first livebirth (such as preeclampsia, preterm birth, or intrauterine growth restriction) and the risk of unexplained stillbirth.	retrospective cohort study 133,163 women having a second birth Scotland	Subsequent pregnancy outcomes	Small for gestational age birth weight (smallest 10% for sex and gestation) shows an increase in the risk of stillbirth in the second pregnancy (HR 2.32 CI 95% 1.82, 2.96 *p* < 0.001)	Risk of explained and unexplained stillbirth in the second pregnancy	Strong

In childhood, having a low birth weight (< 2.5 kg) was associated with an increased likelihood of asthma and wheeze disorders (OR 1.57, 95% CI 1.1-2.25) [38] and moreover was identified as a factor that is often associated with preventable childhood death occurring before the age of five [46].

Preventing low birth weight may be important for some factors affecting cardio vascular health in adulthood. For instance, for every additional kilogram in birth weight, a significant decrease was found in blood pressure, both in childhood (age 4) and throughout adulthood (to age 71) [40]. Similarly increasing birth weight to a normal level was associated with lower cholesterol levels in adulthood [45] though no significant effects on fibrinogen levels in adulthood [41] or diabetes in childhood [43] were noted. Improving birth weight could thus potentially bring about a substantial potential benefit given the associations between high blood pressure and various health conditions such as hypertension and stroke.

Psychological health in adulthood may be affected by low birth weight, with both men and women being more likely to report a history of depression [39], and being of greater risk of suicide [42]. Having a first child with a low birth weight was identified as a significant risk factor for complications in subsequent pregnancies, most notably stillbirth (HR 2.32, 95% CI 1.82-2.96, *p* < .001) [44].

#### Cognitive development and educational attainment

Table 5 presents the findings relating to low birth weight and development and attainment outcomes. Data were extracted from 4 studies examining the effects of low infant birth weight on cognitive development and well-being. Low birth weight may increase the likelihood of requiring special educational support in childhood, with teachers being more likely to recommend specialist support at age 16 for children who had a low birth weight compared to children that fell within the normal range (4.9% vs 2.3%) [47]. This study also showed that low birth weight children were less likely to be in the top performing 15th percentile of their class (13% vs 20%, *p* < 0.01) possibly reflecting differences in cognitive abilities. Indeed, one large scale retrospective cohort study reported that children of low birth weight were almost 2.5 times more likely to experience intellectual impairments (OR 2.67 CI 2.41-2.96), sensory problems (OR 2.85, 95% CI 2.04–3.99) and motor problems (OR 2.47, 95% CI 1.82–3.37) [48].

**Table 5:** Low birth weight and child cognitive/educational outcomes

Study	Aim	Outcome measure	Methods	Results	Notes	Quality assessment
Strauss 2000 [47]	To determine the long-term functional outcome of Small for Gestational Age (SGA) (2436 g) infants	Educational and employment attainment	Prospective cohort study 14,189 full-term cohort infants	Teachers were less likely to rate SGA children in the top 15th percentile of the class at 16 years (13% vs 20%, *P* < 0.01) and more likely to recommend special education (4.9% vs 2.3%, *p* < 0.01). Adults (26 years) born SGA were less likely to have professional or managerial jobs (8.7% vs 16.4%, *p* < 0.01) and reported significantly lower levels of weekly income.		Strong
Mackay et al. 2013 [48]	Association between gestation and birth weight and each cause of special educational need	Causes of special educational needs	Retrospective cohort study 407,503 school children aged between 4 and 19 years	Low birth weight was associated with sensory (OR 2.85, 95% CI 2.04–3.99), physical or motor problems (OR 2.47, 95% CI 1.82–3.37), and intellectual impairments (OR 2.67, 95% CI 2.41–2.96).	No details about weight in grams	Moderate
Bartley et al., 1994 [59]	Association between birth weight and socioeconomic disadvantage during childhood, adolescence, and early adulthood up to 23 years old	Socio-economic disadvantage	Longitudinal analysis 4321 participants have data on birth weight and financial problems; 3370 have data on birth weight, housing conditions and social class. 1958-1981 (when cohort members were followed up at 23 years old)	Birth weight under 2721 g (6 lb) experienced the combined disadvantage of lower social class and overcrowding in the household (*P* = 0 01, 0 01, and 0 13 at ages 7, 1 1, and 16 years, respectively). Lower social class without household amenities or sharing them (*P* = 0.008, 0.002, and 0.18 at ages 7, 11, and 16 years, respectively). Strong association (*P* < 0 001), with cohort members of low birth weight being more likely to experience housing inadequacy.	Statistical analyses were unadjusted	Moderate
O’Brien et al. 2004 [60]	To investigate the neurodevelopmental progress in a cohort of preterm (median 1282 g) survivors by comparing the results of detailed assessment at 8 and 14 years.	Disability and educational outcomes	Longitudinal 151 (out of 224 eligible infants) were available for the assessment at 14-15 years.	In preterm was an increase in the proportion of subjects with disability from 11% at 8 to 22% at 14–15 years of age. Full scale IQ decreased from 104 to 95 from childhood to adolescence, and more adolescents (24%) were requiring extra educational provision than they had at the age of 8 years (15%).	Without control group	Weak

In adulthood, low birth weight children were found to be less likely to have professional or managerial jobs at age 26 (8.7% vs 16.4%, *p* < 0.01) and yield significantly lower levels of weekly income, earning on average £21 per week less than children of normal birth weight (*p* < .01) [47].

### Short-duration to second pregnancy

Table 6 presents the findings relating to a short interval to second pregnancy.

**Table 6:** The consequences of a short interval (< 2 years) to subsequent pregnancy following birth

Study	Aim	Methods	Outcome measure	Findings	Notes	Quality assessment
Smith et al., (2003) [49]	To determine whether a short interval between pregnancies is an independent risk factor for adverse obstetric outcome.	Retrospective cohort study conducted in Scotland, UK. 89,194 women were included	Outcomes were measured for the second child: Intrauterine growth restriction, extremely preterm birth, moderately preterm birth and perinatal death.	A short inter-pregnancy interval (< 6 months) was an independent risk factor for extremely preterm birth (adjusted odds ratio 2.2, 1.4 to 3.6), moderately preterm birth (1.6, 1.3 to 2.0), and neonatal death unrelated to congenital abnormality (3.6, 1.2 to 10.7).	Good range of covariates accounted for in statistical analysis	Moderate

Only 1 study of moderate quality was identified pertaining to the impact of a short duration to a second pregnancy [49]. This study employed a retrospective cohort of 89,194 families in Scotland and focused on the outcomes for the second child. A short inter-pregnancy interval of 6 months or less was identified as an independent risk factor for both extremely preterm birth occurring at 24-32 weeks (OR 2.2, 95% CI 1.4-3.6) and moderately preterm birth occurring at 33-36 weeks (OR 1.6, 95% CI 1.3-2.0). More severely, a short inter-pregnancy interval was also associated with an increased likelihood of neonatal death (unrelated to a congenital abnormality) (OR 3.6, 95% CI 1.2-10.7).

### A&E and outpatient attendances in early childhood and child outcomes

No papers were identified examining the long-term consequences of A&E and outpatient attendances (for any reason) for early childhood.

## Discussion and conclusion

This review aimed to identify, evaluate and summarise all relevant existing studies set within a UK context that have explored the association between the primary outcomes of the Building Blocks trial and longer term effects for the children in terms of health, education, employment and criminality with the view of informing an extrapolation exercise. A broad search was employed which aimed to identify studies relating to maternal smoking during pregnancy, effects of low birth weight, effects of short interval to subsequent pregnancy (< 2 years) and to identify relevant longitudinal studies relating to childhood A&E attendances and inpatient admissions.

After undertaking a rigorous search of the literature guided by pre-defined inclusion and exclusion criteria, 43 studies were identified for inclusion in the review. Of the four trial outcomes of interest, maternal smoking during pregnancy and the effects this has on children’s health and general development yielded the most results. This accounted for 28 of the included studies. Studies centring on outcomes relating to low birth weight were the second most prevalent (14 studies), whereas studies relating to the final two trial outcomes were prominently absent with only one study discussing short interval to subsequent pregnancy and no studies addressing the long-term outcomes associated with early A&E attendance and outpatient attendances, in particular for child injuries and ingestions. This reflects a gap in the UK-based literature within this subject area.

Of the Building Blocks outcomes addressed in the review maternal smoking during pregnancy was most consistently associated with negative child outcomes, particularly health. Weight related problems and child respiratory conditions such as asthma and wheeze were strongly associated with maternal smoking during pregnancy and several other child health outcomes were also highlighted as potential consequences. In terms of societal consequences, one study reported an increased likelihood of convictions for criminal activity associated with children of prenatal smoking mothers [34]. A high cost is associated with criminal activity, particularly that associated with incarceration, with the annual average cost for each prisoner in the UK exceeding £35,000 [50]. Thus if maternal prenatal smoking is linked to children committing criminal offences in later life, this represents another opportunity for later cost savings associated with a reduction in smoking. The limited UK-based literature available suggests that the potential value of reducing prenatal smoking in mothers on children’s educational outcomes is small, with a reduction unlikely to bring about any considerable benefits or cost-savings over the long-term. However, it is important to recognise that this finding is based on only a small number of studies conducted exclusively within a UK context.

Low birth weight was also associated with a vast range of health outcomes such as cardiovascular, respiratory and psychological health. Some potential links with educational attainment were also visible in the literature.

The quality of research entered into a systematic review is directly related to the quality and validity of the results. All included studies were thus assessed for methodological quality using recognised screening criteria [12]. Of the 43 studies included in the review, 41 met the criteria to be considered high or moderate quality, having larger sample sizes, more robust assessment measures and more rigorously conducted statistical analyses controlling for a good range of important potential confounders. Only 2 studies did not meet these criteria. Given that 95% of included studies were considered good quality, we can assume validity in the review findings.

A systematic review was considered the best methodology to answer the current research question. A rigorous search strategy and distinct inclusion and exclusion criteria were employed which yielded a diverse range of relevant good quality studies. The search strategy itself was derived using expert guidance from a systematic review specialist, to ensure all relevant search terms were covered and all relevant databases were searched, thus we can be confident of its adequacy in addressing the question and retrieving the maximum number of results. Pre-defined inclusion and exclusion ensured a robust study selection procedure, further enhanced by the engagement of two reviewers in the selection process.

Examining outcomes pertaining to aspects beyond the realms of health care, such as educational attainment and criminal activity, is a particular strength of the study providing a more complete picture of the potential long-term outcomes associated with the primary Building Blocks trial outcomes. This allows for potential benefits not only to the health care system but also to other areas of society to be observed.

As no studies exploring the association between childhood A&E attendances and admissions were identified, this could highlight a potential limitation in the search strategy. Given that A&E attendances and hospital admissions was considered in the Building Blocks trial to be a proxy for levels of child maltreatment, the inclusion of literature examining the long-term outcomes associated with maltreatment were considered. However, maltreatment is an extremely broad topic and it would not have been feasible to examine the literature within the timeframe of the trial, though has been reviewed elsewhere [51]. Instead, the search aimed to identify studies specifically exploring A&E attendance and hospital admission related studies. The search also aimed to identify papers relating to the long-term consequences of hospital attendances due to child injury and ingestions, though no studies was identified. This reflects a gap in the UK-based literature.

No studies exploring the association between this outcome measure and later health and development were found. This is not a surprising finding given that studies are more likely to report long-term outcomes based on the cause of A&E attendances/hospital admissions, rather than focusing on the dichotomy of whether children experienced these attendances or not. It would be impossible to derive a search strategy that could encompass all causes of A&E attendances/hospital admission and all the long-term consequences. This is thus a limitation of the present review.

For pragmatic reasons, specifically time and capacity, the search strategy was restricted to identify UK-based research only. It is likely that a much larger evidence base exists when the scope is extended beyond the UK, however, this review sought to identify useable evidence to enable modelling of specific short-term outcomes associated with the Building Blocks trial using the most relevant setting of the UK. Had the Building Blocks trial shown a significant effect on prenatal smoking or birthweight, the review would have yielded sufficient information for an extrapolation exercise to be undertaken. Extrapolation would not have been possible for the remaining outcomes, interval to second pregnancy and hospital attendances. Were the effects of the intervention on these outcomes to have been positive, it may have been necessary to consider literature from non-UK sources for extrapolation; however, given that no significant differences were observed for any of the primary outcomes in the trial, this was not required.

This review highlights a number of areas where large potential benefits could be observed as a consequence of improvements made to the primary outcomes of the Building Blocks trial. For instance a reduction in maternal smoking during pregnancy would likely result in a decrease in the proportion of health problems in their children, as well as bringing about gains in educational attainment and criminal activity.

Taken together, the findings of the systematic review show that if improvements could be made in terms of the primary trial outcomes, real benefits could be observed over the longer-term. The most promising gains lie in childhood and adult health, particularly for respiratory illness and weight management problems. Further potential benefits to educational attainment through improved behaviour and cognitive development were also identified.

## Abbreviations

EPHPP: Effective Public Health Practice Project
FNP Family: Nurse Partnership
HTA: Health Technology Assessment
NICE: The National Institute for Health and Care Excellence
RCT: Randomised controlled trial
SIDS: Sudden Infant Death Syndrome
SRU: Social Research Unit
UK: United Kingdom
US: Unites States of America
WSIPP: Washington State Institute for Public Policy
